# Preeclampsia complicated with hypofibrinogenemia: 2 case reports and review of the literature

**DOI:** 10.1186/s12884-023-05965-z

**Published:** 2023-09-01

**Authors:** Shiguang Li, Yanhui Jin, Yanmin Gong, Xia Luo

**Affiliations:** Department of Obstetrics and Gynecology, Qilu Hospital of Shandong University, Jinan, 250000 China

**Keywords:** Preeclampsia, Hypofibrinogenemia, Postpartum haemorrhage (PPH), Heparin

## Abstract

**Background:**

Preeclampsia complicated with hypofibrinogenemia is a rare disorder. We report two cases of severe preeclampsia complicated with hypofibrinogenemia followed by postpartum haemorrhage (PPH).

**Case:**

Two women diagnosed as preeclampsia and hypofibrinogenemia developed severe PPH after undergoing Cesarean sections. Besides supplement with fibrinogen concentrate and supportive treatment, the second patient got administration of heparin after delivery and bleeding was stopped. The haemorrhage in case 1 didn’t disappear until an hysterectomy. The two patients both recovered and were discharged soon.

**Conclusions:**

Severe preeclampsia patients with hypofibrinogenemia could suffer PPH. It’s necessary to detect and master coagulation function. Heparin could be considered to balance hypercoagulation and hypocoagulation to avoid catastrophic haemorrhage and hysterectomy.

## Background

Preeclampsia complicates approximately 3–5% of all pregnancies and is a major cause of maternal and perinatal mortality and morbidity. It is a multi-system disorder originating from maternal vascular endothelial injury and hypertension during gestation. Sometimes preeclampsia is complicated with haematological disorders (e.g., platelet count < 150,000 platelets per µL, disseminated intravascular coagulation (DIC), or haemolysis) [1]. Few articles concentrated on the fibrinogen level in preeclampsia and mostly showed a tendency for a higher level than a normal pregnancy [2–5].

Fibrinogen is essential in platelet aggregation. Hypofibrinogenemia can be caused by heterozygous mutations, liver diseases, or many acquired coagulopathies. The most common symptom was bleeding, including mucocutaneous, soft-tissue, joint, genitourinary, traumatic and surgical, and heavy menstrual bleeding. Laboratory features of hypofibrinogenaemia include prolonged prothrombin (PT), activated partial thromboplastin (APTT) and thrombin clotting (TCT) times and absent or reduced fibrinogen activity [6]. The causes of hypofibrinogenemia in pregnancy mainly included acute fatty liver of pregnancy, placental abruption, congenital hypofibrinogenemia and Kasabach-Merritt syndrome [7–10].

Here, we report two Chinese cases of severe preeclampsia complicated with hypofibrinogenemia followed by PPH. This study conforms with the Declaration of Helsinki. The patients got informed consent, and their anonymity was preserved.

## Case presentation

### Case 1

A 30-year-old woman, gravida 3 para 1 (with histories of full-term delivery and first-trimester pregnancy loss), was admitted to the Qilu Hospital of Shandong University, Jinan, China, at 39^+ 3^ weeks of gestation in March 2018. Blood pressures (BPs) and antenatal examinations were always regular during gestation. She complained of mild contractions and a little vaginal bleeding and was diagnosed with severe preeclampsia ten days ago in the local hospital. Besides hypertension and proteinuria, the patient had mild chest tightness and wheezy breathing for about 30 days. She had no signs of cervical dilatation after vaginal examination, thrombocytopenia, cerebral symptoms, or other complications. The systolic blood pressure (SBP) and diastolic blood pressure (DBP) exceeded 160mmHg and 110mmHg, respectively. The fibrinogen level measured 1.8 g/L, lower than three months ago (3.68 g/L). The patient denied relevant family history.

Routine blood tests showed fibrinogen of 1.82 g/L, HGB of 105 g/L, platelet of normal value, PT and APTT of normal values. Fetal heart rate tracing (FHRT) showed no abnormality. Considering the severity of preeclampsia, we performed a Cesarean section under general anaesthesia after treatment with fibrinogen concentrate (1 g). A male newborn was delivered and weighed 3300 g. Apgar scores were ten at the 1st and 5th minute after birth. There were no signs of placental abruption during the surgery. The contraction was always weak, and continuous bleeding began, which did not improve after treatment with oxytocin (20U, intravenous drip) and misoprostol (400 mg, per rectum). The PPH was first evaluated to be 1000 ml in the hour after cesarean. Repeated blood tests showed HGB of 80 g/L and fibrinogen of 1.26 g/L. Emergency treatment includes uterine package and supplement with red blood cells (4U), fresh frozen plasma (4U), and cryoprecipitate (40U).

Nevertheless, soon after the uterine package, her SBP suddenly dropped to 60mmHg, and oxygen saturation (SaO_2_) fell below 70%. The contraction was still weak with prolonged haemorrhage. The tracheal cannula with 100% oxygen was applied. Further emergency treatment included anti-shock treatment (dexamethasone, metaraminol, and epinephrine), fluid infusion and supplement with red blood cells (8U), fresh frozen plasma (8U), and platelets (1U). Her vital signs recovered approximately 20 min later. The amount of blood loss was secondly estimated to be 3500mL. A repeated coagulation test indicated a fibrinogen level of 1.2 g/L. BPs and SaO_2_ collapsed again when contractions were intensified with haemorrhage still. Finally, a hysterectomy was performed. Then the patient’s vital signs stabilized, and coagulation function improved. On the seventh day after Cesarean, she was discharged with fibrinogen at 3.45 g/L.

### Case 2

A 35-year-old woman, gravida 2 para 1 (with histories of normal full-term delivery and first-trimester pregnancy loss), was admitted to our hospital at 38^+ 3^ weeks of gestation in November 2021. BPs and antenatal examinations were always regular during gestation. The patient complained that the fibrinogen level (2.54 g/L) was lower than five weeks ago (3.53 g/L), and her urine protein was 3 + with SBP of 147mmHg and DBP of 95mmHg. There was no contraction, abdominal pain, vaginal bleeding, vaginal discharge, chest tightness or anxiousness. The patient denied family heredity of coagulation disorders. After admission, we kept continuous observation of BPs and FHRT. Her BPs were unstably elevated. The SBP varied from 140 to 170mmHg, and DBP varied from 90 to 110mmHg. Labetalol (100 mg orally q6h) was administrated; however, there was no obvious improvement. The fibrinogen level measured 2.22 g/L.

Furthermore, reexamination on the next day showed that fibrinogen level was 2.14 g/L and urine protein was 2+. The patient was treated with an emergent Cesarean because of continuous hypertension and decreasing fibrinogen level. Before the surgery, the patient complained of continuous low back pain and a strong sense of contraction. Physical examination showed a uterus with large tension. We suspected this situation as placental abruption and operated immediately under general anaesthesia. A 3300 g female newborn was delivered with the umbilical cord cut off. After airway clearing, stimulus, and oxygen inhalation, the baby was kept warm. Apgar scores were 8 and 10 at the 1st and 5th minute after birth.

After the Cesarean, her vital signs were regular and stable. However, there was continuous bleeding at a small amount even after administration of oxytocin (20U, intravenous drip) and misoprostol (400 mg, rectal usage). So we kept regularly-repeated coagulation tests, as shown in Table 1. The fibrinogen level was 1.23 g/L (10:19 am) soon after the Cesarean. So the patient received emergency treatment, including transfusion of heparin (20IU/mL, intravenous drip, 14drops/min), erythrocytes (8U), platelet (1U), plasma (650mL), fibrinogen concentrate (12 g), albumin (10 g) and meropenem (1 g). After heparin administration, the fibrinogen level gradually increased and reached 3.09 g/L at 2:07 pm, bleeding decreased, and her vital signs were stable. Other supportive treatments contained administration of misoprostol (400 mg) per rectum and injection of carboprost tromethamine (250 µg) into the myometrium.

**Table 1:** Perioperative coagulation test parameters

	8:00	10:19	10:58	12:01	12:22	13:05	14:07	14:52	16:30
PLT	150	85	86	114	112	100		90	82
APTT	28.2	32.6	32	32.8	34.9	36.3	32.5	30.3	30.2
fibrinogen	2.14	1.23	1.76	2.3	2.48	2.53	3.09	3.02	2.96
DDI	3.27	33.51	42.56	44.51	42.97	42.13	34.14	29.3	23.91
TAT	72.2	944.57	521.29	298.16	239.64	162.2	111.06	74.95	66.07
PIC	8.28	20.97	26.22	27.97	25.74	31.83	25.41	24.93	25.41
t-PAI·C	5.48	5.4	4.7	3.24	3.52	4.38			
TM	5.53	4.24	4.68	5.98	5.92	6.48			
TAT/PIC	8.72	45.04	19.88	10.66	9.31	5.09	4.37	3	2.6

Abbreviations: PLT, platelet (×10^9^/L), APTT, activated partial thromboplastin time (s), DDI, D-dimer (g/L), TAT, thrombi-antithrombin complex (ng/mL), PIC, plasmin-a2-antiplasmin complex (TU/mL), t-PAI·C, tissue plasminogen activator inhibitor complex (ng/mL), TM, thrombomodulin (µg/mL), TAT/PIC (ng/TU).

On the first day after cesarean, BPs were around 133/76mmHg. Reexamination of the blood test showed HGB of 70 g/L, platelet of 80 × 10^9/L, fibrinogen of 3.36 g/L and albumin of 31.9 g/L. The patient received a transfusion of erythrocytes (2U) to correct anaemia. The BPs on the second day after delivery measured 130–150/70-90mmHg, so labetalol (100 mg orally q6h) was administrated. The BPs did not decrease, so the dosage of labetalols was adjusted to 200 mg orally tid, and nifedipine controlled-release tablets (30 mg orally bid) were supplemented. Then BPs approximately returned to normal coverage on the fifth-day post-delivery. The patient and the newborn were discharged on the seventh day after delivery without obvious discomfort.

## Discussion

We reported two cases of severe preeclampsia complicated with hypofibrinogenemia. The main problems of the two cases were the significant decrease of fibrinogen and continuous hypertension. After excluding family heredity and previous history, we diagnosed the cases as hypofibrinogenemia.

The fibrinogen levels are physiologically increased during gestation and might have a critical effect on implantation and the development of fetal vascular communication [11]. However, this elevation in fibrinogen does not stop possible complications such as antepartum haemorrhage (APH) and PPH in women with hypofibrinogenaemia. Fibrinogen replacement could be necessary to reduce the risk of bleeding and stabilize pregnancy [6].

Preeclampsia has been a serious pregnancy complication and a worldwide health concern for a long time. The exact pathogenesis and mechanism of preeclampsia have not been completely expounded, and various risk factors have been investigated and discussed. It is well known that vascular endothelial cell injury plays a fundamental role in multi-organ injury, resulting in preeclampsia. Abnormal coagulation and bleeding mechanisms are important manifestations [12]. Some clinicians focused on the association between fibrinogen and preeclampsia. Karehed et al. also elucidated that fibrinogen concentration was increased in preeclampsia before gestational week 32 compared to their peer controls [5]. Rijin and his colleagues analyzed secondary-pregnancy clinical characteristics of females after a first pregnancy complicated by early-onset preeclampsia. They revealed that recurrent preeclampsia was relevant with higher pre-pregnancy levels of C-reactive protein and fibrinogen than those without recurrent preeclampsia [2]. Duan et al. found that fibrinogen in mild preeclampsia patients was not less than in controls, and fibrinogen in severe preeclampsia patients was significantly higher than in controls [3]. Another research demonstrated that at the gestational age of 32 weeks, the plasma fibrinogen γ chain levels in preeclampsia patients were significantly elevated [13]. However, Chen and her colleagues concluded that the fibrinogen concentration in preeclampsia patients was significantly lower than in healthy pregnant women (1.59 ± 1.07 versus 4.19 ± 0.63, p < 0.0001). They attributed this difference to the high proportion of patients with severe preeclampsia (80/125, 64%) [14]. The two kinds of conclusions above seem contradictory, which could be explained by the characteristics of different stages in DIC. Treatment with heparin should be considered when thrombosis predominates [15]. If uncontrolled, preeclampsia might progress from local thrombosis to the consumption of clotting substances, including fibrinogen, that is to say, from hypercoagulation to hypocoagulation, leading to hypofibrinogenemia and even post-partum haemorrhage [16, 17]. In case 2, we kept observation of the values of TAT/PIC to evaluate coagulation and fibrinolysis and decided on the administration of heparin, which might prevent bleeding via targeting the stage of hypercoagulation.

## Conclusion

Hypofibrinogenemia in patients with severe preeclampsia could be attributed to DIC. It is important to distinguish different stages in DIC, for example, by the test of TAT and PIC, and prevent further progression. Besides administration of fibrinogen concentrate and other supportive treatment, heparin should be considered to balance hypercoagulation and hypocoagulation to avoid catastrophic haemorrhage and hysterectomy.

## Data Availability

The data that support the findings of this study are available from the corresponding author upon reasonable request.

## Abbreviations

DIC: Disseminated intravascular coagulation
